# Long-Term Calcium Monitoring Post Parathyroidectomy for Primary Hyperparathyroidism

**DOI:** 10.7759/cureus.53591

**Published:** 2024-02-05

**Authors:** William Jenkins, Edward Chisholm, Faith Protts

**Affiliations:** 1 Otolaryngology, University of Bristol, Bristol, GBR; 2 Otolaryngology, Musgrove Park Hospital, Somerset National Health Service (NHS) Foundation Trust, Taunton, GBR; 3 Otolaryngology, University Hospitals, Bristol National Health Service (NHS) Foundation Trust, Bristol, GBR

**Keywords:** hypercalcaemia, calcium monitoring, recurrence, primary hyperparathyroidism, parathyroidectomy

## Abstract

Background: Cure rates following parathyroidectomy for primary hyperparathyroidism are excellent, with well-documented low short-term recurrence rates of hypercalcaemia. Rates of long-term recurrence have been investigated to a lesser extent, but recent studies have reported higher than anticipated rates. This study sought to evaluate recurrence rates at more than four years post seemingly corrective surgery and depending on the findings, propose whether recommendations of annual calcium monitoring post-parathyroidectomy are appropriate based on the limited data available at the time of formulating guidelines.

Methods: Fifty-two sequential parathyroidectomies for primary hyperparathyroidism from 2014-2016 from a single unit were retrospectively followed up with serum calcium levels. A hospital computer system was used to collect data on pre-operative, immediate post-operative and most recent follow-up calcium levels. Patients were excluded if there was no minimum of 48 months between the operation date and most recent calcium. Recurrence was defined as hypercalcaemia more than six months after eucalcaemia post-parathyroidectomy.

Results: Of the 52 cases analysed, two were lost to long-term follow-up, two patients died during the follow-up period while 10 did not meet the inclusion criteria of at least 48 months follow-up. This resulted in a cohort of 38 patients (mean age 66.4 years, 78.9% female). The median follow-up of 73.17 months (range 48.77-95.47 months) demonstrated a hypercalcaemia recurrence of 5.26% (2/38 patients). These cases were due to misdiagnosed parathyroid hyperplasia as opposed to suspected adenoma. Therefore, the long-term cure rate was 94.74% (36/38 patients).

Conclusion: These findings support the high cure rates and low recurrence rates of the numerous short-term studies already performed despite a longer follow-up period. This is in contrast to recent series which have documented a higher recurrence in the long-term. This study would, therefore, suggests recommendations of annual calcium monitoring are excessive and that less frequent calcium monitoring is necessary in the first few years post-operation. However, the 5.26% recurrence rate in this study is not insignificant and follow-up is still paramount. Therefore, following the initial post-operative assessment, the authors propose a follow-up at the five-year mark and an annual continuation from this point forward due to the evidenced delayed recurrence of hypercalcaemia.

## Introduction

Primary hyperparathyroidism is an endocrine condition in which the chief cells of a single parathyroid gland or multiple glands produce excessive parathyroid hormone (PTH) [1]. This most frequently occurs as the result of an adenoma affecting a singular parathyroid gland, which is benign in nature in over 85% of cases [2]. Other causes include parathyroid gland hyperplasia, multiple adenomas, carcinoma and multiple endocrine neoplasia (MEN) type 1 and 2a [1]. The majority of patients remain asymptomatic (70-80%), but symptomatic presentations are mainly related to hypercalcaemia [1] and include lethargy, bone pain, nephrolithiasis, polyuria, and polydipsia, constipation, cardiovascular disease and psychological symptoms [1,3]. The condition is typically characterised by inappropriately high PTH levels in the presence of high serum calcium levels, i.e., hypercalcaemia, due to the activation of osteoclasts increasing calcium levels via bone resorption [1]. Primary hyperparathyroidism is a major cause of hypercalcaemia, with a prevalence of one to four per 1,000 people [3] and most commonly affects post-menopausal women [1].

Surgical management, in the form of parathyroidectomy, is the definitive treatment [2]. The surgical approach has progressed from traditional four-gland exploration to more focused minimally invasive parathyroidectomy, especially in the case of a single overactive adenoma, in more recent times [4]. This is evidenced by shorter operating times, reduced rates of hypocalcaemia post-operatively, less post-operative pain, and quicker recovery and discharge, in addition to the aesthetic benefit of a smaller scar [5]. Curative success is usually defined as eucalcaemia (serum calcium adjusted for albumin in the range of 2.2-2.6 mmol/L) [3], for at least six months post-parathyroidectomy [6], and this occurs in circa 95% of cases [4]. A role for PTH monitoring in representing cure post-parathyroidectomy has also been proposed due to 23-44% of patients having elevated PTH levels despite eucalcaemia [6].

Recurrence is defined as hypercalcaemia (serum calcium adjusted for albumin ≥2.6 mmol/L) occurring after six months of eucalcaemia (serum calcium adjusted for albumin in the range of 2.2-2.6 mmol/L) post seemingly corrective parathyroidectomy [2]. Recurrence may occur due to misdiagnosed multiple adenomas or parathyroid gland hyperplasia, ectopic glands, genetic and environmental factors, as well as possible parathyroid malignancy [7]. Short-term recurrence rates suggest concordance with cure rates; one study demonstrated a recurrence rate of 2.5% over a 9.2-month median follow-up [8], while a systematic review demonstrated rates of 1.6% over a mean follow-up duration of 33.5 months [2]. However, fewer series have investigated the long-term recurrence rates of hypercalcaemia, and there is suspicion these rates may be higher. One of which demonstrated a recurrence rate as high as 14.8% over a median follow-up duration of 9.2 years [7], thus casting doubt over whether high rates of short-term normalisation of serum calcium could be extrapolated over longer periods of follow-up.

The aim of this study is to evaluate long-term recurrence rates of hypercalcaemia post-parathyroidectomy in comparison to the well-established low rates of short-term recurrence. This would also aid in assessing the suitability of the 2019 National Institute for Health and Care Excellence's (NICE) proposed guidelines for annual serum calcium tests post-parathyroidectomy [3]. Whilst these guidelines were introduced following the operation dates of this dataset, the findings of this study could shed light on whether annual monitoring is an appropriate follow-up (in the context of high rates of hypercalcaemic recurrence) or whether (if low long-term rates of recurrence) it is in fact of clinical value and cost-effective to monitor levels as regularly.

This article was previously presented as an oral poster presentation at the 6th Congress of European Otorhinolaryngology-Head and Neck Surgery (ORL-HNS) from October 29 - November 2, 2022, in Milan, Italy.

## Materials and methods

A retrospective study on follow-up calcium monitoring in patients post-parathyroidectomy was conducted. The cohort was selected from a database of all patients who underwent parathyroidectomy for primary hyperparathyroidism by a single surgeon at Musgrove Park Hospital, Taunton, between 2014 and 2016. This data was retrieved from the Pan-Surgical Electronic Logbook database of parathyroidectomies performed in this time period by a single Ear, Nose, and Throat Consultant Surgeon. All patients undergoing parathyroidectomy were for primary hyperparathyroidism.

Data was collected on the closest pre-operative serum calcium levels available for each patient (which were typically elevated), the most immediate post-operative calcium levels available (which were typically within the reference range of 2.2 to 2.6 mmol/L due to corrective surgery) and the most recent calcium values available. Patient demographics were also documented. This was all collected from the hospital’s MAXIMS^TM^ Order Communications and Result Reporting System (IMS MAXIMS, Dun Laoghaire, Dublin). From the data collected, the percentage of patients with hypercalcaemia pre-operatively and the mean calcium value were then calculated along with the median duration between the operation date and the most recent calcium result, which formed the follow-up time. Data was then presented in a spreadsheet (included in Table 1) which included the following headings: operation date; patient’s age (years); patient’s sex; pre-operative calcium level (mmol/L); initial post-operative calcium level (mmol/L); most recent calcium level (mmol/L) and duration in months between operation date and most recent calcium level.

**Table 1:** Data collection spreadsheet Asterisks denote excluded cases. F = Female; M = Male

Operation Date	Patient Age (Years)	Sex	Pre-Operative Calcium Level (mmol/L) and Date Performed	Initial Post-Operative Calcium Level (mmol/L) and Date Performed	Most Recent Calcium Level (mmol/L) and Date Performed	Duration Between Operation and Most Recent Calcium Level (Months)
*16/01/2014 00:00	75	F	2.47 (28/10/13)	2.42 (17/01/14)	No result available	N/A
16/01/2014 00:00	72	M	2.81 (24/09/13)	2.47 (17/01/14)	2.38 (25/06/21)	89.3
30/01/2014 00:00	66	F	2.8 (10/12/13)	2.61 (07/02/14)	2.57 (26/03/21)	85.87
30/01/2014 00:00	61	F	2.45 (19/08/13)	2.37 (31/01/14)	2.48 (25/05/21)	87.83
11/02/2014 00:00	58	F	2.89 (10/02/14)	2.37 (24/02/14)	2.54 (06/02/19)	59.87
12/02/2014 00:00	72	F	2.69 (04/02/14)	2.49 (13/02/14)	2.77 (04/09/20)	78.77
10/03/2014 00:00	61	F	2.73 (16/09/13)	2.29 (21/03/14)	2.46 (28/05/21)	86.6
10/03/2014 00:00	61	M	2.88 (03/02/14)	2.26 (03/04/14)	2.50 (20/08/21)	89.33
17/03/2014 00:00	67	F	2.87 (07/01/14)	2.27 (19/03/14)	2.33 (31/03/19	60.47
03/04/2014 00:00	72	M	2.9 (24/02/14)	2.28 (07/05/14)	2.36 (24/12/20)	80.7
14/04/2014 00:00	66	F	2.62 (08/10/13)	2.31 (05/05/14)	2.45 (29/04/21)	84.5
14/04/2014 00:00	75	F	2.63 (27/03/14)	2.19 (02/05/14)	2.30 (23/12/20)	80.3
02/06/2014 00:00	62	F	2.65 (30/12/13)	2.18 (06/06/14)	2.22 (16/05/22)	95.47
24/06/2014 00:00	85	F	2.8 (09/10/13)	2.17 (06/08/14)	2.31 (07/02/21)	79.47
18/09/2014 00:00	62	F	2.89 (24/06/14)	2.46 (19/09/14)	2.50 (17/05/22)	91.97
16/10/2014 00:00	57	F	2.79 (05/09/14)	2.45 (29/10/14)	2.48 (01/04/19)	53.5
16/10/2014 00:00	75	F	3.18 (18/06/14)	2.5 (20/10/14)	2.27 (29/07/21)	81.43
*06/11/2014 00:00	89	M	3.01 (18/07/14)	2.15 (21/11/14)	2.17 (15/08/17) Deceased 04/2019	33.3
10/11/2014 00:00	81	F	2.91 (09/09/14)	2.36 (02/12/14)	2.37 (18/01/19)	50.27
*26/11/2014 00:00	58	F	2.72 (01/09/14)	2.31 (09/12/14)	2.31 (06/02/18)	38.37
29/01/2015 00:00	57	F	2.78 (03/07/14)	2.36 (04/02/15)	2.29 (14/06/20)	64.53
19/02/2015 00:00	72	M	2.8 (25/11/14)	2.4 (08/03/15)	2.52 (05/05/22)	86.53
13/03/2015 00:00	53	F	3.01 (27/11/14)	2.52 (27/03/15)	2.52 (12/05/21)	73.97
*13/05/2015 00:00	83	F	2.84 (01/05/15)	2.28 (19/05/15)	2.31 (24/03/16) Deceased 03/2016	10.37
01/06/2015 00:00	58	F	2.96 (18/12/14)	2.5 (22/06/15)	2.51 (22/08/19)	50.7
18/06/2015 00:00	60	M	2.87 (17/03/15)	2.63 (19/06/15)	2.21 (22/10/21)	76.13
02/07/2015 00:00	78	M	3.2 (18/02/15)	2.52 (04/07/15)	2.34 (30/12/21)	77.93
06/08/2015 00:00	74	F	3.01 (03/07/15)	2.29 (20/08/15)	2.24 (12/11/20)	63.2
19/09/2015 00:00	57	F	3.06 (14/07/15)	2.3 (25/09/15)	2.35 (31/12/20)	63.4
01/10/2015 00:00	78	F	3.05 (08/09/15)	2.38 (07/10/15)	2.41 (08/03/21)	65.23
29/10/2015 00:00	73	M	2.73 (05/06/15)	2.52 (17/11/15)	2.70 (08/02/22)	75.33
09/11/2015 00:00	61	F	2.61 (15/09/15)	2.38 (10/11/15)	2.33 (20/12/21)	73.37
*10/11/2015 00:00	63	M	2.89 (18/06/15)	2.49 (16/12/15)	2.45 (18/08/17)	21.27
03/12/2015 00:00	86	F	2.74 (21/10/15)	2.22 (14/12/15)	2.40 (01/01/22)	72.97
25/01/2016 00:00	60	F	2.81 (19/01/16)	2.43 (05/02/16)	2.36 (05/11/21)	69.37
*22/02/2016 00:00	64	F	2.6 (29/12/15)	2.34 (03/03/16)	No result available	N/A
03/03/2016 00:00	69	F	2.74 (23/12/15)	2.38 (18/03/16)	2.40 (04/11/21)	68.03
*08/03/2016 00:00	71	F	2.89 (11/11/15)	2.24 (22/03/16)	2.28 (17/07/17)	16.3
*29/03/2016 00:00	59	F	2.57 (01/03/16)	2.28 (31/03/16)	2.3 (14/02/19)	34.53
*11/04/2016 00:00	69	F	2.89 (21/01/16)	2.36 (06/05/16)	2.41 (16/11/16)	7.17
14/04/2016 00:00	71	F	2.81 (01/10/15)	2.46 (06/05/16)	2.30 (07/05/20)	48.77
*10/05/2016 00:00	60	F	2.80 (02/09/15)	2.45 (23/05/16)	2.44 (15/02/18)	21.17
27/06/2016 00:00	81	F	2.65 (09/11/15)	2.19 (06/07/16)	2.45 (09/02/22)	67.43
30/06/2016 00:00	64	F	2.75 (06/05/16)	2.30 (01/07/16)	2.51 (07/10/21)	63.23
29/07/2016 00:00	65	F	2.65 (17/06/16)	2.38 (05/08/16)	2.38 (21/07/21)	59.73
04/08/2016 00:00	58	F	2.66 (25/02/16)	2.08 (02/09/16)	2.40 (27/07/21)	59.73
*30/08/2016 00:00	68	F	2.74 (24/05/16)	2.49 (21/11/16)	2.30 (01/10/18)	25.03
15/09/2016 00:00	67	F	3.22 (06/09/16)	2.49 (17/09/16)	2.55 (21/01/22)	64.2
14/11/2016 00:00	28	M	2.67 (08/09/16)	2.45 (17/11/16)	2.45 (02/02/22)	62.63
*28/11/2016 00:00	61	M	3.13 (31/10/16)	2.22 (02/12/16)	2.38 (01/08/17)	8.13
*28/11/2016 00:00	62	F	2.76 (11/10/16)	2.13 (14/12/16)	2.25 (24/04/19)	28.87
*28/11/2016 00:00	74	F	2.63 (27/06/16)	2.39 (13/12/16)	2.47 (01/10/19)	34.1

Patients were excluded if there was no long-term biochemical data available, which included a complete set of pre-operative, initial post-operative, and most recent calcium values. The most recent calcium value needed to be taken more than 48 months post-parathyroidectomy in order to examine possible long-term recurrence. These parameters formed the inclusion criteria for the study.

Residual hypercalcaemia was defined as elevated serum calcium levels (≥ 2.6 mmol/L) within six months of operation, while recurrence was defined as elevated serum calcium levels (≥ 2.6 mmol/L) after more than six months post-operation following a previous period of eucalcaemia (2.2-2.6 mmol/L). Therefore, the cure was defined as eucalcaemia (2.2-2.6 mmol/L) more than six months following parathyroidectomy. The rates of recurrence formed the primary outcome of the study.

## Results

Pre-operative characteristics

**Figure 1:**
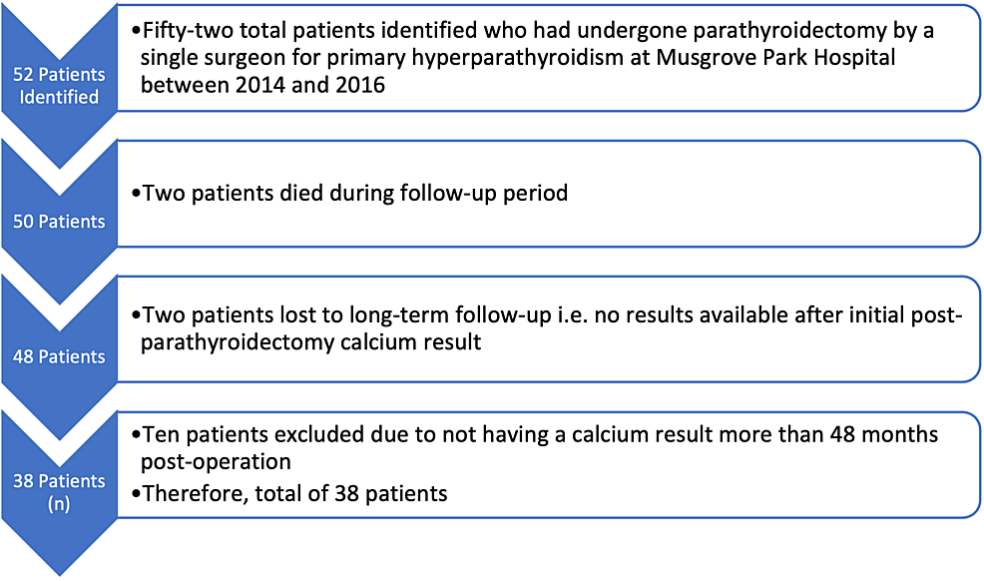
Exclusion criteria flowchart Process demonstrating the exclusion of 14 patients from the original cohort of 52 to reach a total of 38 patients (n).

The database consisted of 52 patients who presented for surgical treatment of primary hyperparathyroidism at Musgrove Park Hospital, Taunton, between 16th January 2014 and 28th November 2016. From this original database, 14 patients in total were excluded due to unsuitable length of follow-up, two of which were due to unrelated deaths during the follow-up period, before the minimum of 48 months post-operation. A further two patients were lost to follow-up and never had a calcium reading recorded after the initial post-operation result. The remaining 10 patients did not meet the threshold of having a calcium result available at least 48 months post-parathyroidectomy. This resulted in a total cohort of 38 patients (n); all of which had pre-operative, initial post-operative and most recent calcium results (over 48 months post-operation) available. This process is demonstrated in Figure 1.

The mean age of the patients in this study was 66.4 years old (range 28 to 86 years) and there was a 78.9% female predominance. A total of 37 patients (97.37%) had hypercalcaemia pre-operatively (defined as serum calcium adjusted for albumin ≥ 2.6 mmol/L) with a mean calcium value of 2.82 mmol/L (range 2.45 to 3.22 mmol/L). The remaining patient (2.63%) was eucalcaemic (defined as serum calcium adjusted for albumin in the range of 2.2 to 2.6 mmol/L) hyperparathyroid with advanced osteoporosis being the indication for operative management. These pre-operative characteristics can be visualised in Table 2. 

**Table 2:** Pre-operative patient characteristics Demographics of the 38 patients and pre-operative calcium levels with rates of hypercalcaemia and eucalcaemia

Variable	Value
Mean age (years), (range)	66.4 (28-86)
Female (%)	78.9
Mean pre-operative serum calcium (mmol/L), (range)	2.82 (2.45-3.22)
Pre-operative hypercalcaemia (≥2.6 mmol/L) (%)	97.37
Pre-operative eucalcaemia (2.2-2.6 mmol/L) hyperparathyroid (%)	2.63

Outcomes

A total of 35 patients (92.11%) had eucalcaemia (2.2 to 2.6 mmol/L) immediately post-operatively. Two patients (5.26%) had hypercalcaemia (≥ 2.6 mmol/L), while one patient (2.63%) had hypocalcaemia (<2.2 mmol/L). The hypocalcaemic patient later became eucalcaemic shortly after and the original hypocalcaemia was likely due to a lag before physiological adjustment to parathyroidectomy. This was likely corrected by supplemental calcium. The mean calcium value immediately post-operation was 2.37 mmol/L (range 2.08 to 2.63 mmol/L). The 5.26% of patients with initial post-operative hypercalcaemia were not deemed to have residual hypercalcaemia as, at follow-up, their levels had normalised to within the reference range (2.2 to 2.6 mmol/L). These immediate post-operative outcomes can be visualised in Table 3. 

**Table 3:** Immediate post-operative outcomes Mean calcium level immediately post-operatively with rates of hypercalcaemia, hypocalcaemia and eucalcaemia.

Variable	Value
Mean immediate post-operative serum calcium (mmol/L), (range)	2.37 (2.08-2.63)
Immediate post-operative hypercalcaemia (≥ 2.6 mmol/L) (%)	5.26
Immediate post-operative hypocalcaemia (<2.2 mmol/L) (%)	2.63
Immediate post-operative eucalcaemia (2.2-2.6 mmol/L) (%)	92.11

The duration (in months) between the operation date and the most recent calcium result formed the follow-up period. Having excluded any patients with a follow-up period of less than 48 months, the median follow-up was 73.17 months. The range of follow-up periods was 48.77 to 95.47 months.

At the time of follow-up, 36 patients (94.74%) were eucalcaemic (2.2 to 2.6 mmol/L), and as these results were all taken more than six months post-parathyroidectomy, this was the cure rate. Two patients (5.26%) were deemed to have recurrent hypercalcaemia (≥2.6 mmol/L). Both patients were misdiagnosed with parathyroid adenoma pre-operatively (despite positive pre-operative ultrasound and Sestamibi parathyroid imaging) but, following four gland explorations and histology, were found to have parathyroid hyperplasia as the underlying aetiology. These patients opted for no further surgical intervention; thus their calcium levels remained elevated. No patients ultimately had residual hypercalcaemia due to normalisation at follow-up. The mean calcium value at the time of follow-up was 2.42 mmol/L and the range was 2.21 to 2.77 mmol/L. These follow-up outcomes can be visualised in Table 4.

**Table 4:** Follow-up outcomes The median duration of follow-up and mean follow-up calcium level with rates of hypercalcaemia and eucalcaemia.

Variable	Value
Median follow-up duration (months), (range)	73.17 (48.77-95.47)
Mean follow-up serum calcium (mmol/L), (range)	2.42 (2.21-2.77)
Follow-up hypercalcaemia (≥2.6 mmol/L) (%) i.e., recurrence rate	5.26
Follow-up eucalcaemia (2.2 to 2.6 mmol/L) (%) i.e., cure rate	94.74

## Discussion

This study reports a 5.26% recurrence rate after seemingly corrective parathyroidectomy. In this series, 100% of the recurrences occurred more than six years (78.77 and 75.33 months, respectively) after the surgical intervention, thus demonstrating the possibility of recurrence beyond the scope of the previous series’ short-term follow-ups.

Whilst the recurrence rate in this study (5.26%) is greater than the mean figure of 1.6% reported in a systematic review of recurrence rates following minimally invasive parathyroidectomy over a mean follow-up of at least a year [2], it is markedly lower than the 14.8% 10-year recurrence rate [7] and the 10.7% recurrence up to 17 years post-operation [9] reported in other long-term studies. It is important to appreciate that one of the long-term studies [7] found that over one-third of recurrences occurred over 10 years post-operation and considering the median follow-up in this study was circa six years, it is possible the recurrence rate of 5.26% may increase if further follow-up continued.

The cases of recurrence in this series were due to misdiagnosed parathyroid hyperplasia as opposed to suspected adenoma. This was confirmed by histology in both cases. A longer-term study attributed recurrences to de novo disease due to unusually long duration of eucalcaemia prior to elevation of calcium levels [9]. Due to the shorter follow-up period in this study, it was not possible to explore such possibilities.

Standard practice at the time patients in this study had their operation was to monitor initial serum calcium levels post-operatively and if within the reference range of 2.2 to 2.6 mmol/L (as was the case in 92.11% of patients in this study), discharge the patient; assuming there were no other postoperative complications or comorbidities affecting recovery. In 2019, NICE guidelines introduced a recommendation for annual calcium checks post-parathyroidectomy [3]. The results from this study would suggest this is possibly an overly regular check-up as there were no cases of residual hypercalcaemia, all the recurrences occurred after six years post-operation, and this is supported by the two other long-term studies referenced [7,9]. A more time and cost-effective guidance would be to defer follow-up to five years post-operation (assuming no residual hypercalcaemia prior to hospital discharge or at the post-operation clinic) and then introduce annual calcium monitoring from this point onwards, as the results suggest this is the lag time before recurrence occurs. This is a unique UK-based study as there is a paucity of research into the recurrence of hypercalcaemia at this longer-term follow-up, and the design of this study should ensure the potential for reproducibility on a larger scale in the future. 

Limitations

This study is not without its limitations. The retrospective nature of data collection meant a large proportion of the original cohort had to be excluded due to an inadequate length of follow-up. In addition, the follow-up threshold for inclusion was set at an arbitrary length of 48 months, and a later cut-off may have influenced the recurrence rate due to the seemingly delayed presentation of primary hyperparathyroidism recurrence typically. A prospective study over a pre-determined length of time would lead to standardised intervals of calcium monitoring, thus allowing for easier interpretation. The regularity of calcium monitoring was also impacted by the fact these operations were undertaken several years prior to NICE’s 2019 guidelines for annual check-ups.

These findings are based on using hypercalcaemia as the sole marker for the recurrence of hyperparathyroidism and neglecting to consider PTH as an additional indicator. PTH levels are not frequently monitored post-operatively and thus are an additional flaw of a retrospective study. A recent clinical review evidenced elevated PTH levels in up to 46% of eucalcaemic patients post-parathyroidectomy [10]. However, as 97.4% of patients in this study were hypercalcaemic pre-operatively, elevated calcium levels seem to be a suitable proxy of hyperparathyroidism. Despite this surrogate being deemed appropriate, considering PTH in addition to calcium would undoubtedly lead to a more accurate rate of recurrence. For this reason, the recurrence rate of 5.26% in this study may be an underestimate of the true recurrence in this cohort.

The typical patient demographic for parathyroidectomy means that long-term follow-up is often less achievable. This is supported by the mean age of 66.4 years in this cohort and the fact that two patients were excluded because of death due to other morbidity.

It would be naïve to ignore the impact coronavirus disease 2019 (COVID-19) has had on both general practice (GP) and outpatient appointments. This has undoubtedly led to less frequent calcium check-ups, which as well as leading to more patients being excluded from the study due to inadequate length of follow-up, has also possibly masked some recurrences.

Finally, this data was collected from a list of patients undergoing parathyroidectomy in a single hospital by a single surgeon during a period of only three years. Therefore, such results will certainly be influenced by individual surgical experience and expertise as well as hospital factors. It is difficult to predict whether the results drawn from this study would be replicated on a larger scale.

## Conclusions

In summary, the 5.26% recurrence rate in this study is not insignificant, and follow-up is still paramount due to the often asymptomatic, but potentially life-altering morbidity associated with the recurrence of primary hyperparathyroidism. It is thus essential that patients are safety-netted by means of education on potential symptoms of recurrence to ensure these cases are diagnosed and rectified. However, this study has demonstrated the excellent cure rates of parathyroidectomy and the low recurrence rates in the initial years following operative intervention. This study would, therefore, suggest recommendations of annual calcium monitoring are excessive and that less frequent monitoring is necessary in the first few years post-operation. Therefore, following the initial post-operative assessment, the authors propose a follow-up at the five-year mark and an annual continuation from this point forward due to the evidenced delayed recurrence of hypercalcaemia and the paucity of evidence regarding recurrence rates beyond this study's follow-up.
